# Using pay for performance incentives (P4P) to improve management of suspected malaria fevers in rural Kenya: a cluster randomized controlled trial

**DOI:** 10.1186/s12916-015-0497-y

**Published:** 2015-10-16

**Authors:** Diana Menya, Alyssa Platt, Imran Manji, Edna Sang, Rebeccah Wafula, Jing Ren, Olympia Cheruiyot, Janice Armstrong, Brian Neelon, Wendy Prudhomme O’Meara

**Affiliations:** Moi University School of Public Health, College of Health Sciences, Eldoret, Kenya; Duke Global Health Institute, Duke University, Durham, NC USA; Academic Model Providing Access to Healthcare, Eldoret, Kenya; Moi University School of Medicine, College of Health Sciences, Eldoret, Kenya; Department of Medicine, Duke University, Durham, NC USA

**Keywords:** Kenya, Malaria, Malaria case management, Pay for performance, Performance-based incentives

## Abstract

**Background:**

Inappropriate treatment of non-malaria fevers with artemisinin-based combination therapies (ACTs) is a growing concern, particularly in light of emerging artemisinin resistance, but it is a behavior that has proven difficult to change. Pay for performance (P4P) programs have generated interest as a mechanism to improve health service delivery and accountability in resource-constrained health systems. However, there has been little experimental evidence to establish the effectiveness of P4P in developing countries. We tested a P4P strategy that emphasized parasitological diagnosis and appropriate treatment of suspected malaria, in particular reduction of unnecessary consumption of ACTs.

**Methods:**

A random sample of 18 health centers was selected and received a refresher workshop on malaria case management. Pre-intervention baseline data was collected from August to September 2012. Facilities were subsequently randomized to either the comparison (n = 9) or intervention arm (n = 9). Between October 2012 and November 2013, facilities in the intervention arm received quarterly incentive payments based on seven performance indicators. Incentives were for use by facilities rather than as payments to individual providers. All non-pregnant patients older than 1 year of age who presented to a participating facility and received either a malaria test or artemether-lumefantrine (AL) were eligible to be included in the analysis. Our primary outcome was prescription of AL to patients with a negative malaria diagnostic test (n = 11,953). Our secondary outcomes were prescription of AL to patients with laboratory-confirmed malaria (n = 2,993) and prescription of AL to patients without a malaria diagnostic test (analyzed at the cluster level, n = 178 facility-months).

**Results:**

In the final quarter of the intervention period, the proportion of malaria-negative patients in the intervention arm who received AL was lower than in the comparison arm (7.3 % versus 10.9 %). The improvement from baseline to quarter 4 in the intervention arm was nearly three times that of the comparison arm (ratio of adjusted odds ratios for baseline to quarter 4 = 0.36, 95 % CI: 0.24–0.57). The rate of prescription of AL to patients without a test was five times lower in the intervention arm (adjusted incidence rate ratio = 0.18, 95 % CI: 0.07–0.48). Prescription of AL to patients with confirmed infection was not significantly different between the groups over the study period.

**Conclusions:**

Facility-based incentives coupled with training may be more effective than training alone and could complement other quality improvement approaches.

**Trial registration:**

This study was registered with ClinicalTrials.gov (NCT01809873) on 11 March 2013.

**Electronic supplementary material:**

The online version of this article (doi:10.1186/s12916-015-0497-y) contains supplementary material, which is available to authorized users.

## Background

Overtreatment of fevers with antimalarials is a major global health challenge that has been described across malaria-endemic countries around the world. In developing countries, in a typical rural health center, more than 90 % of febrile patients are treated with antimalarials when a diagnostic test is not available [1] and even when testing is available, 40–80 % of patients with a negative malaria test still receive an antimalarial [2–9]. Nationwide in Kenya, 20 million treatment courses of artemether-lumefantrine (AL, the first-line antimalarial in Kenya) were dispensed through government health facilities in 2009, compared to only 9 million cases of reported malaria.

Overuse of antimalarials poses a significant health risk to both present and future patients and puts a financial strain on the health system. Unnecessary consumption jeopardizes the useful therapeutic life of first-line therapies by accelerating the spread of drug resistance [10, 11] and threatens the sustainability of donor-subsidized drug procurement programs.

Since 2010, both the Government of Kenya and the World Health Organization (WHO) [12] recommend diagnostic testing before treatment with antimalarials, but this recommendation is often ignored in practice. In the current system in Kenya, as in many countries in sub-Saharan Africa, there is little or no incentive for the patient or the clinician to adhere to the test-then-treat policy for malaria. Antimalarials are free to the facility and to the patient, whereas antibiotics are often dispensed for a small fee. In addition, laboratory diagnosis of malaria costs the patient $0.50 USD, more than it costs to buy subsidized first-line antimalarials such as AL over the counter in a retail shop. This combination of perverse incentives contributes to the overtreatment of fevers with antimalarials and poor compliance with the test-then-treat policy.

Presumptive treatment of fevers with antimalarials is an example of systemically poor provider behavior that may be amenable to change using pay for performance (P4P) incentives. Such extrinsic incentives could overcome the perverse incentive structure described above. Performance-based incentives have an established role in health care delivery in the developed world and have been implemented extensively [13–16]. However, there is limited evidence to support such incentive programs in health systems of developing countries. A handful of studies have shown promise, but methodologically rigorous evaluation of P4P, particularly experimental evidence from randomized trials, is lacking [17–23].

We tested a novel strategy to introduce facility-based incentives in order to increase diagnostic testing, improve appropriateness of prescription practices and increase adherence to malaria diagnostic test results. There are key differences in our P4P strategy compared to previous approaches. First, previous P4P approaches in Africa emphasized volume of patients receiving specific services, whereas our incentives are focused around quality of malaria case management. Second, we used facility-directed incentives rather than payments to individual providers, thereby supporting health system infrastructure and emphasizing the teamwork required across the departments (laboratory, pharmacy, records and clinical services) for good case management.

We describe the results of a cluster randomized controlled trial to measure the impact of facility-directed P4P incentives to improve management of malaria in rural health facilities. The intervention was targeted to the health facility, therefore a cluster design using the health facility as the unit of randomization was chosen. We report the impact of incentives on three key outcomes, the first two at the individual level and the third at the cluster level: 1) inappropriate treatment of malaria-negative patients with artemisinin-based combination therapies (ACTs); 2) appropriate treatment of confirmed malaria cases with ACTs; and 3) laboratory testing before treatment with ACTs.

## Methods

### Study setting and facility enrollment

The study was carried out within the catchment area of the Academic Model Providing Access to Healthcare (AMPATH), which includes 17 districts in the former Western Province and northern Rift Valley Province^1^ of Kenya. Malaria endemicity varies across the study area: the Western Province experiences high transmission, whereas the Rift Valley has little or no transmission but is prone to epidemics when climate conditions are suitable.

**Fig. 1:**
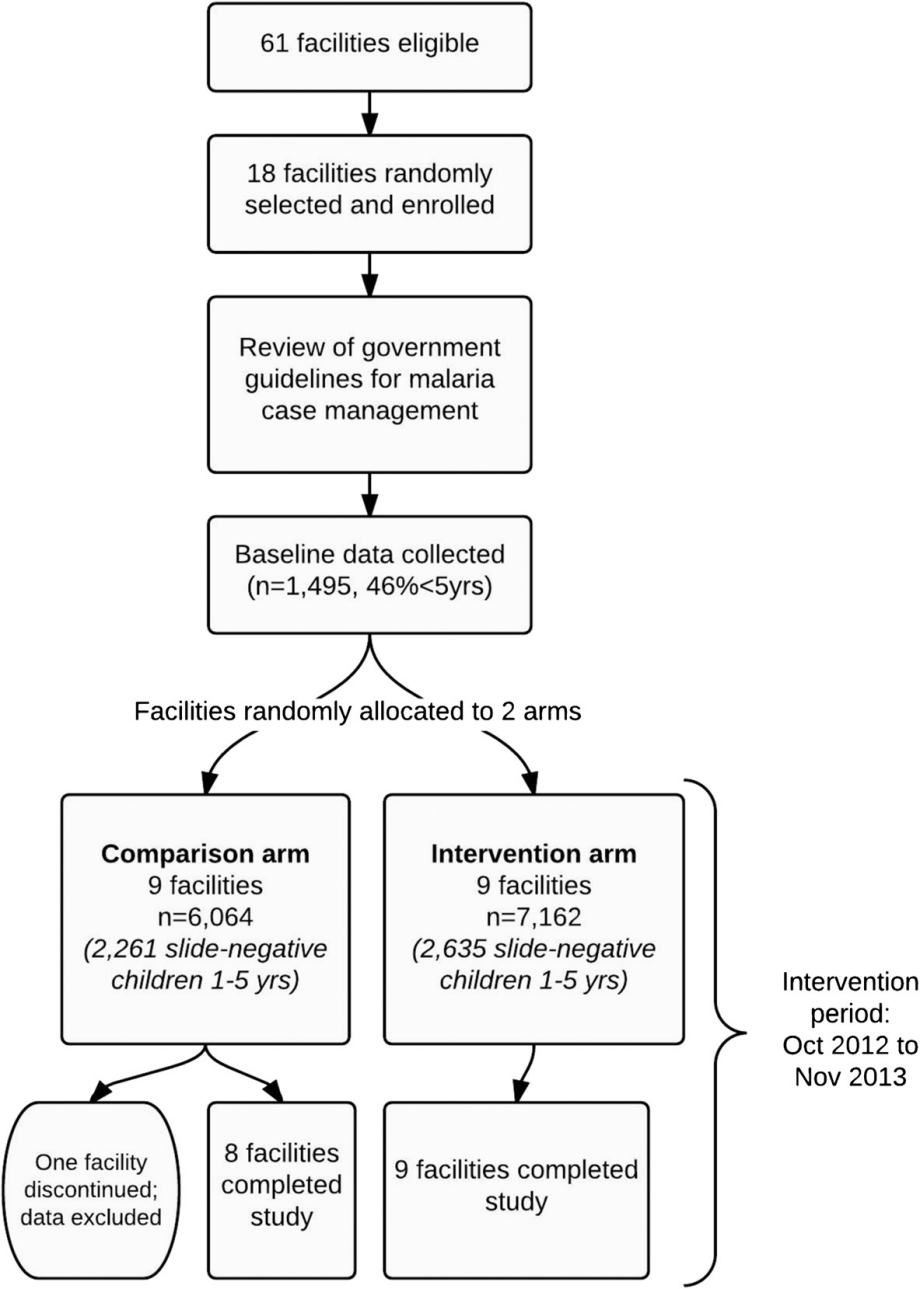
Diagram of study enrollment, randomization and analysis

The unit of enrollment and randomization was the health center. Selection of facilities occurred in two phases: 1) random sampling to identify 18 eligible facilities; and 2) random assignment of the sampled facilities to the two arms (Fig. 1). All government-owned health centers in the 17 districts were eligible for the study if they had capacity to parasitologically diagnose malaria. Only government health facilities were eligible, since only government facilities receive Health Sector Services Funds (HSSF). Hospitals were not included because they receive referrals and severe or complicated cases and their HSSF allocation is much higher than lower-level facilities, all of which could affect the response to the intervention.

There were 61 eligible health centers; 18 were selected by simple random sampling. We chose facilities by simple random sampling rather than probability proportional to size because our intention was to describe facility behavior from a representative sample of facilities, not the underlying patient population attending facilities. Nine facilities were from areas designated by the Division of Malaria Control as high transmission; the remaining were from low epidemic-prone areas [24]. One facility was dropped from the study after a prolonged absence of the laboratory technician. Although it was eligible at enrollment, the technician was absent for 2.5 of the first 4 months and eventually fired in month 6. They were no longer able to offer diagnostic testing and therefore were not able to complete the study.

At the time the study was initiated, malaria rapid diagnostic tests (RDTs) were not available in government facilities. Therefore, we focused on facilities that could confirm suspected malaria by microscopy. We deemed it unethical to emphasize withholding antimalarials from malaria-negative patients without ensuring that the quality of diagnosis was sufficiently high. One laboratory technician from each facility attended a training course at the Malaria Diagnostic Center in Kisumu, Kenya [25]. In order to monitor quality of diagnosis, all study facilities participated in a monthly external quality assurance program, the details of which can be found in Wafula et al. [26]. Sensitivity and specificity of malaria diagnosis was included as a performance indicator for intervention facilities.

Dissemination of the *National Guidelines for the Diagnosis, Treatment and Prevention of Malaria in Kenya* [27] by the Division of Malaria Control began in late 2010 and included training and distribution of job aides. It was important to ensure that the government malaria treatment policies were reviewed with study facilities to avoid the perception that ‘test-then-treat’ guidelines were specific to our study. Therefore, the guidelines were reviewed in a brief 1-day workshop prior to randomization to arms.

### Randomization to intervention arm

Following training, enrolled facilities were randomly allocated to the two arms. Assignment was performed in an open forum with attendees from each clinic and district and a representative from the Division of Malaria Control. Randomization was done by blinded draw where each facility within a district had an equal probability of allocation to either arm [28].

### Incentive intervention

We tested the use of facility-directed, performance-based incentives to improve quality of care for suspected malaria fevers. Facilities in the intervention arm received incentive payments based on seven performance indicators. Performance indicators, and the value assigned to each indicator and performance level, are shown in Table 1. They included recording of patient identification numbers, quality of laboratory diagnosis and clinician adherence to the laboratory diagnosis. The incentives were designed to foster cooperation between departments (pharmacy, laboratory and clinical care) and none were punitive. Spending of the incentive funds was subject to certain restrictions according to the requirements of the government’s HSSF: they could only be used by the facility for equipment, supplies, repairs and basic labor [29]; and not for payments to individual clinicians or employees. Harmonization of the incentive program and the HSSF was critical for establishing whether P4P could be incorporated into the HSSF.

**Table 1:** Indicators for calculating incentives, weights and values assigned to performance levels

	Indicator	Weight	Scale
1	Percent of laboratory registry entries with patient number (all patients)	5 %	100 % - full marks
			>90 % - 60/100
			>85 % - 50/100
			<85 % - 0/100
2	Percent of AL registry entries with patient number (all patients)	2.5 %	100 % - full marks
			>90 % - 60/100
			>85 % - 50/100
			<85 % - 0/100
3	Percent of antibiotic registry entries with patient number (all patients)	2.5 %	100 % - full marks
			>90 % - 60/100
			>85 % - 50/100
			<85 % - 0/100
4	Sum of sensitivity and specificity	20 %	>190 - full marks
			>170 - 80/100
			>150 - 60/100
			<150 - 0/100
5	Percent of patients given AL without a malaria test (slide or RDT)	30 %	<5 % - full marks
			5–10 % - 70/100
			11–20 % - 50/100
			>20 % - 0
6	Percent of patients with a positive malaria test given AL	15 %	100 % - full marks
			>90 % - 70/100
			>80 % - 50/100
			>70 % - 25/100
			<70 % - 0/100
7	Percent of patients with a negative malaria test given AL	25 %	<5 % - full marks
			5–10 % - 70/100
			11–20 % - 50/100
			>20 % - 0

The scale indicates what percent of the money for that indicator is awarded at each performance level. For example, if less than 100 % but at least 90 % of patient records had a patient number, then 60 % of the money for that indicator was awarded. AL, artemether-lumefantrine; RDT, rapid diagnostic test

Incentive amounts were calculated and communicated quarterly during routine facility visits. Facilities could earn a maximum of $1,175 USD per quarter (100,000 KSh). This amount was estimated to be approximately equivalent to the amount of money that could be saved if overuse of ACT was curbed. After communication of the incentive amount earned, facilities submitted a budget to use their incentive allocation and the study team executed the budget. Supplies and equipment were procured by the study team and delivered to the facility. Funds were disbursed for repairs, construction or labor costs. Every effort was made to disburse incentive funds within 4 weeks.

### Data collection

Data were collected during monthly visits from August 2012 until November 2013 (pre-intervention baseline data from September 2012 to October 2012, intervention period from October 2012 to November 2013). A member of the District Health Management Team (DHMT) accompanied the data collection team on all facility visits. Data were collected by capturing electronic images of standard, government-issued facility registers in use at all the facilities. A patient’s data were eligible for inclusion in the study if they either: 1) had a laboratory test for malaria; or 2) received AL. AL is the only ACT available in government health facilities. Infants and pregnant women are often prescribed quinine, therefore women who were seen in antenatal clinic or otherwise indicated as pregnant, and children less than 1 year of age were excluded. This allowed us to focus specifically on AL prescription.

Our sample was based on a systematic random sampling of patients tested for malaria with either microscopy or RDT (the latter were sporadically available in some facilities after the third month of the intervention). The appropriate sampling interval ‘n’ was calculated based on the number of malaria tests performed that month and the expected proportion of children and negative tests. We sampled every nth patient from the laboratory register resulting in a sample of 26 malaria-negative children per month per facility (see sample size calculation below) and a variable number of adults and slide-positive patients depending on the proportion of malaria-negative children seen at the facility.

Each patient sampled from the laboratory register was then traced in the clinical register and the pharmacy registers (antibiotics register and AL register) to determine which diagnoses were assigned and which drugs were prescribed. Drug prescriptions were taken from both the pharmacy register and clinical register due to incomplete pharmacy record keeping in some facilities. In addition, AL registers were reviewed and 6–7 random days each month were matched to the laboratory register to determine which patients received AL without visiting the laboratory.

Antimalarial drug stocks were monitored throughout the study. The study was prepared to provide AL to any facility in either arm with a shortage. AL was provided to two facilities in mid-2013 for a 1-month window. There were no stockouts in any study facilities.

Monthly facility reports were downloaded from the Kenya Health Information System [30]. Missing data was supplemented with copies of monthly reports kept at the facility when possible.

### Power and analysis

We calculated power to estimate our primary endpoint in an important subgroup (children under 5 years of age). We based our calculation on a cluster randomized, difference-of-proportions test with the primary outcome being the proportion of slide-negative patients receiving AL at the end of the intervention period. Based on data from a representative facility, we estimated that an average facility would see 300 sick children per month, at least 30 % of which would be tested for malaria and 50–90 % test negatively. If 50 % of slide-negative children in the control arm receive AL, then 18 facilities (nine per arm) with 26 slide-negative children per facility would give 90 % power to detect a minimum difference of 0.15 in the proportion of negative children receiving AL. We assumed a two-sided test with a 95 % significance level and an intracluster correlation (ICC) of 0.002. Assuming an ICC of 0.005 reduced our power slightly to 88 %. We intended to analyze the data longitudinally in order to identify a burn-in phase or changes in response to the incentives over time, therefore we planned to sample approximately 26 slide-negative children per facility in each of the 13 months of the study (1 month of baseline plus 12 months of intervention), for a total of 6,084 observations of children under 5 years of age with a negative test.

We powered our study to measure the outcomes in children because, prior to the revised 2010 malaria treatment guidelines, national policy advised treating children less than 5 years of age presumptively with antimalarials for any fever. The guidelines issued in 2010 changed the policy to parasitological confirmation before treatment for all age groups, including children under 5 years of age. We expected that ‘test-then-treat’ guidelines would not be adopted very readily for this age group and thus could be a behavior that was amenable to change under extrinsic incentives. However, the largest proportion of wasted ACTs (ACTs given to slide-negative patients) occurs in adults. Therefore, both age categories were important for the study.

For the primary endpoint of AL prescription to malaria-negative patients, we used a mixed-effects logistic regression model of individual patients with random intercepts for each facility. The effect of time on the outcome and interaction with the intervention was modeled using dummy variables for baseline and each quarter. This was chosen based on model fit and interpretability, since the intervention incentives were calculated quarterly. Covariates incorporated into the final model included age group (<5 years and ≥5 years of age) and gender of the patient, average volume of slides read in the facility in the previous year, use of RDT or slide for diagnosis, and location in a high or low transmission zone. We used the same approach to evaluate our secondary endpoint of AL prescription to slide-positive patients.

ICC is reported for each model. ICC was also calculated for the comparison between the arms at the final time point (month 12) to provide an estimate more comparable to our power calculation. These are provided as supplementary material (Additional file 1).

An important secondary endpoint was the rate of AL prescriptions to patients without a malaria test. This endpoint was evaluated at the cluster level. The number of AL prescriptions dispensed to patients without a test was calculated for each facility in each month of the study. The effect of the intervention on AL prescribed to untested patients was estimated with a negative binomial regression model of the number of patients prescribed AL without a test, with an offset equal to the log of the total patient volume.

Finally, we calculated the difference in risk differences from baseline to quarter 4 between the intervention and control arms using results from the unadjusted mixed-effects model assuming that random effects are zero (Additional file 2).

### Funding and ethical review

This study was supported by the National Institute of Allergy and Infectious Diseases (NIAID) of the National Institutes of Health (NIH) (R21 AI095979). The protocol was reviewed and approved by the Duke University Institutional Review Board and the Moi University Institutional Research and Ethics Committee (IREC/2012/18 approval # 000804). Permission and participation of the Ministry of Health and the Division of Malaria Control was secured prior to the initiation of the study. Consent was sought from the Provincial Health Management Teams (Western Province and Rift Valley Province), each DHMT and the officer in charge of each study facility.

## Results

### Study facilities

All study activities took place between June 2012 and December 2013. Baseline data was collected in September and October 2012. The intervention was launched in October 2012 and continued until October 2013.

In total, 18 facilities were enrolled and 17 facilities (nine intervention and eight comparison) completed the 1-year intervention phase of the study. One facility was excluded from the analysis due to extended absence of a laboratory technologist and inability to provide laboratory diagnosis of malaria. The remaining facilities completed the study and were included in the analysis (Fig. 1). Overall, 14,721 patient encounters (including 5,584 slide-negative children aged 1–5 years) were included in the patient-level analysis.

There were no significant differences in demographics of the patients attending facilities or the slide positivity rate in each arm (Table 2). Comparison facilities had higher baseline patient volume in the laboratory than intervention facilities, but the difference was not statistically significant (*P* = 0.24).

**Table 2:** Facility and patient characteristics in each arm

Characteristics	Intervention (n = 9)	Comparison (n = 8)
Facility		
High transmission	5 of 9	4 of 8
Number of clinical staff at baseline (median, range)	6 (3–11)	7 (4–9)
Number of laboratory staff at baseline (median, range)	1.5 (1–3)	1 (1–2)
Average slides read per month (SD)	240.0 (126.9–353.1)	372.4 (135.0–609.7)
Percent positive slides	17.5 % (0.7–27.8)	19.1 % (1.3–28.0)
Percent of diagnoses by RDT	12.3 % (1.3–45.5)	11.1 % (0.0–47.4)
Sensitivity (SD)	93.70 (89.04–98.35)	99.47 (98.95–99.98)
Specificity (SD)	89.35 (83.40–95.31)	88.63 (80.56–96.70)
Patient		
Number of patient observations	8,045	6,894
Age (SD)	16.6 (15.9–17.4)	14.5 (14.1–14.9)
Children aged 1–5 years	3,650 (45.9 %)	3,250 (48.1 %)
Female	4,656 (57.9 %)	4,067 (59.0 %)

RDT, rapid diagnostic test; SD, standard deviation

### Impact of interventions on facility performance

Raw proportions, unadjusted odds ratios (ORs) and adjusted ORs from the mixed-effects logistic regression model for the prescription of AL to malaria-negative and malaria-positive patients in quarter 4 compared to baseline are shown in Table 3. Full model results including coefficients of all covariates can be found in Additional file 3.

**Table 3:** Mixed-effects logistic regression of AL use by malaria status in quarter 4 compared to baseline. Comparison of AL treatment in intervention and control arm for all patients and by sub-group (transmission zone, age group)

	Raw proportion								
Endpoint	Comparison			Intervention					
	Clusters	Baseline	Quarter 4	Clusters	Baseline	Quarter 4	Unadjusted OR	Adjusted OR	ICC (95 % CI)
							(95 % CI)	(95 % CI)^a^	
Malaria-negative patients receiving AL									
All ages	8	0.16	0.11	9	0.22	0.07	0.34	0.36	0.140
							(0.24–0.53)	(0.24–0.57)	(0.073–0.252)
Aged 1–5 years	8	0.20	0.12	9	0.27	0.10	0.43	0.48	0.135
							(0.24–0.79)	(0.25–0.86)	(0.063–0.262)
Aged 6 years and over	8	0.14	0.10	9	0.19	0.05	0.28	0.27	0.159
							(0.15–0.52)	(0.15–0.52)	(0.081–0.288)
High transmission	4	0.32	0.18	5	0.36	0.12	0.41	0.45	0.109
							(0.25–0.67)	(0.28–0.74)	(0.045–0.241)
Low transmission	4	0.06	0.02	4	0.11	0.01	0.16	0.16	0.112
							(0.05–0.52)	(0.05–0.56)	(0.039–0.281)
Malaria-positive patients receiving AL									
All ages	8	0.71	0.78	9	0.71	0.80	0.88	0.91	0.019
							(0.46–1.69)	(0.47–1.77)	(0.007–0.054)
Aged 1–5 years	8	0.76	0.77	9	0.77	0.82	0.93	0.98	0.029
							(0.35–2.49)	(0.37–2.62)	(0.008–0.101)
Aged 6 years and over	8	0.66	0.79	9	0.64	0.77	0.89	0.87	0.005
							(0.36–2.18)	(0.35–2.15)	(0.0003–0.070)
High transmission	4	0.72	0.85	5	0.79	0.82	0.54	0.54	0.004
							(0.23–1.27)	(0.23–1.28)	(0.0002–0.075)
Low transmission	4	0.71	0.62	4	0.59	0.54	1.10	1.16	0.028
							(0.34–3.54)	(0.36–3.78)	(0.006–0.120)

^a^Estimates adjusted for quarter, age category (except for stratified analysis), gender, mode of diagnosis (RDT or microscopy), transmission zone (except for stratified analysis) and average monthly volume of slides read in the facility in the preceding year. AL, artemether-lumefantrine; CI, confidence interval; ICC, intracluster correlation; OR, odds ratio; RDT, rapid diagnostic test

By the final quarter of the intervention period, prescription of AL to malaria-negative patients in the intervention arm dropped from 22.4 % at baseline to 7.3 % (112/1,526) in quarter 4 compared to a decline of only 5.4 percentage points in the intervention arm (16.3 % to 10.9 %). After correcting for individual and facility covariates, the odds of receiving AL following a negative test in the intervention arm was 0.15 in quarter 4 compared to baseline and 0.42 in the comparison arm relative to baseline, indicating a larger improvement in the intervention arm over the study period. This translates to a 2.75-fold greater reduction in odds of inappropriate prescription of AL in the intervention arm relative to the comparison arm from baseline to the end of the study.

Facilities in high transmission areas had ten times higher odds of AL prescription to negative patients than those in low transmission areas (adjusted OR = 10.36, 95 % CI: 4.96–21.64; Additional file 3), therefore we further stratified our analysis on transmission zone (Table 3). Intervention and comparison facilities in low malaria transmission areas had equally low rates of AL prescription to malaria-negative patients by the end of the study period, but intervention facilities improved more relative to baseline than comparison facilities. In high transmission areas, facilities in both arms dispensed AL to 35 % of malaria-negative patients at baseline. By the end of the study, intervention facilities again showed more improvement than comparison facilities (67 % reduction compared to 50 % reduction in raw proportion, ratio of adjusted OR = 0.45, 95 % CI: 0.28–0.74).

Malaria-negative children under 5 years of age were more likely to get AL than malaria-negative adults in both arms and the odds of a malaria-negative child receiving AL was even higher when diagnosed by microscopy compared to RDT (adjusted OR = 0.77, 95 % CI: 0.60–0.99; Additional file 3).

At baseline and endline, there was no significant difference in prescription practices to malaria-confirmed cases between the intervention and comparison groups. AL dispensed to laboratory-confirmed malaria cases improved slightly, but not significantly, in both arms by the fourth quarter of the study.

The rate of prescription to patients without a test was evaluated at the cluster level. The percent of AL given to patients without a test was 25.7 % in the intervention arm at the end of the study compared to 40.3 % in the control arm. After adjusting for transmission zone and volume of patients seen at a facility, the rate of AL prescriptions to patients without a test was 5-fold lower in the intervention arm (Incidence rate ratio (IRR) = 0.18, 95 % CI: 0.07–0.48; Table 4).

**Table 4:** Effect of intervention on diagnostic testing prior to treatment with AL

n = 174	IRR	95 % CI
Intervention	0.179	0.067–0.478
Standardized patient volume	0.687	0.616–0.766
High transmission	0.021	0.011–0.040

CI, confidence interval; IRR, incidence rate ratio

### Intervention outcome

The maximum amount that could be earned by an intervention facility over the 1-year study period was 400,000 KSh (approximately $5,000 USD). The mean quarterly amount earned was $668 USD, approximately half of the total possible amount. There was considerable variation between facilities in the amount earned, indicating that individual facility characteristics or context were important (by ANOVA, 78 % of variance is explained by between-facility variation and 22 % within-facility, across quarters).

The indicator for which intervention facilities showed the most improvement was the percent of malaria-negative patients who received AL (22.4 % at baseline down to 7.3 % in Q4; Table 5) and percent of patients given AL without a malaria test (41 % down to 26 %). However, the latter still remained high at approximately 26 %. Laboratory performance indicators steadily improved each quarter. Quarterly performance is also shown for the comparison arm.

**Table 5:** Quarterly performance of facilities for incentivized indicators

Facility	Baseline	Quarter 1	Quarter 2	Quarter 3	Quarter 4
Intervention					
Mean incentive earned in USD		$593.4	$726.5	$629.5	$724.9
(SD)		(281.0)	(311.3)	(282.0)	(332.9)
Percent malaria-negative patients given AL	22.4 %	15.8 %	15.0 %	12.0 %	7.3 %
Percent malaria-positive patients given AL	70.8 %	78.3 %	74.0 %	76.9 %	79.6 %
Percent of AL given without a test	-	40.7 %	29.1 %	35.5 %	25.7 %
Sensitivity + Specificity	-	151.8	173.9	182.9	185.8
Comparison					
Percent malaria-negative patients given AL	16.3 %	11.6 %	11.7 %	6.5 %	10.9 %
Percent malaria-positive patients given AL	71.4 %	69.5 %	79.0 %	79.1 %	78.0 %
Percent of AL given without a test	-	57.1 %	46.3 %	45.1 %	40.3 %
Sensitivity + Specificity	-	152.9	183.3	190.6	184.1

-, no baseline information available. AL, artemether-lumefantrine; SD, standard deviation

Twenty-seven and 39 % of incentive funds were spent on laboratory equipment and patient equipment, respectively, including microscopes, delivery beds, centrifuges, steam sterilizers, biosafety cabinet, blood pressure cuffs and minor surgical tools. Seven percent was used for facility repairs such as plumbing, electrical, fencing, security grills and doors. Eleven percent was used for laboratory reagents for urinalysis, blood grouping, blood sugar testing, hemoglobin measurement and other medical consumables.

### Pre-study to post-study differences in clinical diagnosis and AL prescription

**Fig. 2:**
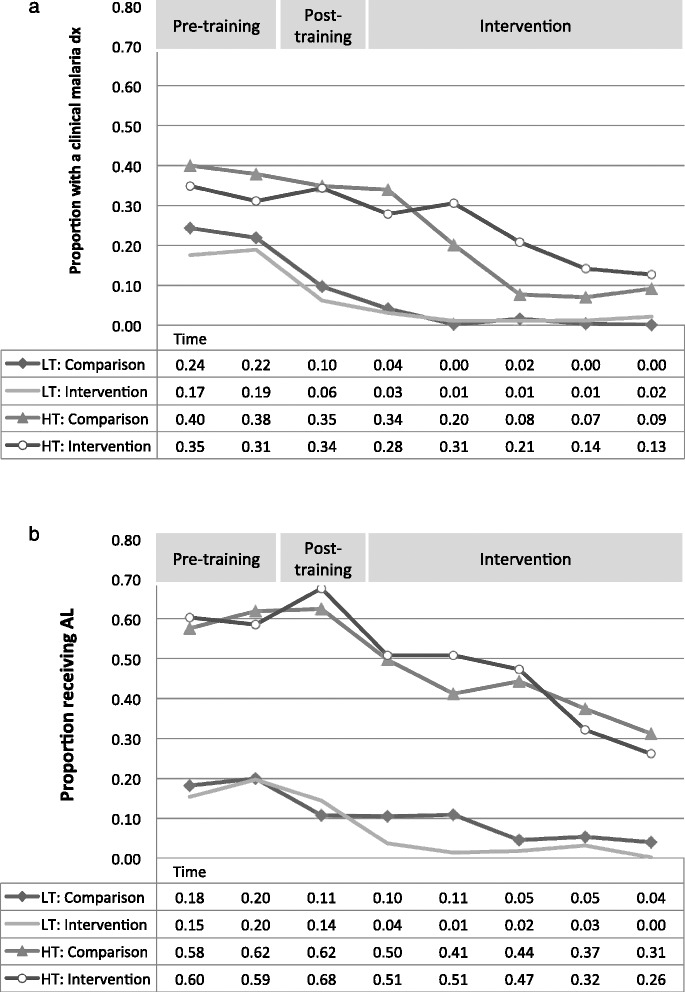
**a** Proportion of total patients attending a facility who received a clinical diagnosis of malaria and **b** proportion of total patients who received AL before training, after training and after randomization to arms. Results are presented by quarter starting in January 2012 and stratified by transmission zone and study arm

Individual patient observations describing malaria testing and treatment were not available prior to the study. Therefore, to observe change in behavior before and after study training (microscopy training and 1-day workshop to review the malaria treatment guidelines with clinicians) compared to the intervention period, we extracted monthly facility-level data from the Kenya Health Information System. The ratio of total monthly cases of malaria diagnosed clinically (without parasitological confirmation) to total outpatients, and the ratio of AL courses dispensed to total outpatients is averaged by quarter and plotted in Fig. 2.

During the 6 months prior to training, about 20 % of all patients in low transmission facilities were given a clinical diagnosis of malaria and about 15 % of all patients were given AL. After training, the proportion receiving a clinical diagnosis of malaria declined by half, but the proportion receiving AL changed only slightly. After randomization to arms, the proportion of patients receiving AL dropped rapidly in the intervention arm, but remained stable in the comparison arm for the first 6 months. A similar pattern is observed in high transmission: before the study, 40 % of patients received a clinical diagnosis of malaria, but 60 % of all patients received AL. AL prescription increased slightly in the 3-month period between training and assignment to arms. Both clinical diagnoses and AL prescription began to decline after the start of the intervention period.

Overall, the training alone seems to have reduced the proportion of patients assigned a clinical diagnosis of malaria in low transmission facilities, but did not substantially change prescription practices in either zone, indicating that AL was being prescribed to patients with other diagnoses. It is only after the intervention began that substantial changes in AL prescription are observed.

These numbers show general trends, but it should be noted that they are presented as raw figures, not adjusted for facility-level covariates. The ratio of clinically diagnosed patients will be dependent on availability of laboratory services (for example, some facilities have laboratory services on the weekend while some do not), which may not be balanced between arms. For the pre-defined study endpoints, we were able to exclude individual dates when laboratory services were not available, which we cannot do for aggregated monthly data. Similarly, AL dispensing depends heavily on the slide-positivity rate and the availability of AL, which also may not be balanced between arms. Prior to the study initiation, there likely were periodic stockouts of AL. Finally, routine monthly reporting data is subject to high rates of missingness.

## Discussion

Results from a cluster randomized study of performance-based incentives in rural government health facilities shows significant improvement in management of suspected malaria fevers over the study period in response to incentives. We observed important and significant improvements in rates of malaria testing prior to treatment and reduction in over-prescription of AL to non-malaria fevers. The reduction in AL prescriptions to malaria-negative patients was observed to some degree in both groups, but the intervention group showed more than a 2.5-fold greater reduction in odds of dispensing AL to slide-negative patients than the comparison group by the final quarter.

The effect of the intervention on AL overuse was primarily observed in the final quarter and there are several possible reasons for the delayed impact of incentives. First, the relatively short intervention period (1 year) may have hindered our ability to measure a steady-state effect of the incentives. Second, it is possible that it took some time for the facilities to understand how to maximize their incentives or to appreciate the additional resources available. Finally, it is also possible that in a system with high staff turnover, providers may be less motivated by facility-based incentives if they anticipate being transferred and are not able to benefit from additional resources to the facility.

The percent of negative patients receiving AL at baseline was lower than expected based on previous studies and may have reduced our ability to measure an earlier impact of incentives. As a result, the absolute difference between the arms at the end of the study was small (3.6 percentage points), although the percent improvement (67 % in the intervention arm) would be remarkable if reproducible at higher starting levels of over-dispensing of AL. We considered whether the pre-intervention training contributed to low AL prescription to malaria-negative cases at baseline. However, recently published nationwide surveys show AL prescription rates of approximately 15 % to malaria-negative patients, which is consistent with our observations at baseline [31]. In addition, Mbacham et al. [32] showed no effect of a 1-day basic training on any aspect of malaria case management 3–6 months after training. Finally, analysis of monthly reports from study facilities before and after training suggests little or no impact of training on clinically diagnosed malaria and AL prescription. Therefore, the impact of pre-study training on the outcome was likely small. If there was an early effect of training on AL prescription in the first few months, the intervention could have been responsible for maintaining and enhancing those improvements in the intervention arm as comparison facilities lost momentum and returned to *status quo*. This would also explain the larger difference between the arms toward the end of the study period.

Another possible factor contributing to initial improvement in both arms may have been the monthly visits to each facility, which could have reinforced the training. A similar effect was seen in a recent trial in Tanzania that measured the impact of RDTs and training on antimalarial prescription [33]. They also saw that overuse of antimalarials remained very low in the control arm throughout the 1-year intervention period, probably reflecting the combination of training plus regular supervision. Although our study was not designed to separately measure the effect of supervision, this distinction may not be relevant since such a program would not be implemented in the absence of similar supervision structure. The effect of the incentive in isolation of the associated framework may not be meaningful. Because of the cluster randomized controlled design, we can confidently attribute the excess improvement in the intervention arm to the incentives.

There was no effect of the intervention on prescription of AL to malaria-positive patients. Other studies of malaria prescription practice have recorded similar low rates of prescription of first-line antimalarials to confirmed malaria cases [31, 34], which persisted under other intervention strategies [32, 33]. The reasons for this are not entirely clear and a better understanding of clinical decision making for confirmed malaria cases is required.

Pay for performance programs have shown mixed results, particularly in health systems of developing countries [23, 35]. Similar to our study, a pilot program in Uganda showed parallel improvement in incentive and non-incentive groups [36]. They pointed to contextual factors that may have undermined the extrinsic motivation provided by the incentives. During our intervention period, the Ministry of Health removed user fees from all peripheral health facilities, including laboratory fees. Although there were no stockouts of AL in study facilities, they struggled under increased patient volume and lack of user fees to replenish supplies. The overall situation eroded morale and may have reduced the impact of extrinsic motivation. Some laboratories simply could not keep up with the number of patients, which could explain why 30–40 % of AL was dispensed to patients without a laboratory test.

The fact that our study relied solely on routine record keeping at the facility is both a strength and a weakness. Using routine government-issued tools ensured that we were measuring behavior within the normal practice and there was no added burden of record keeping and no excessive observer effect that is present with exit interviews. Unfortunately, we found record keeping to be incomplete at some facilities, particularly in the pharmacy, which is often staffed by an untrained casual worker. This may be responsible for the unexpectedly low observed prescription rates to slide-positive patients. However, this was not a systematic bias (that is, affecting the intervention more than the comparison arm, or negative patients more than positive patients) and so is not likely to have biased our results. Low observed prescription rates to malaria-positive patients also suggests that gaming or data manipulation was not an issue in our study. Facilities would have had to orchestrate a high degree of collusion across several departments at the level of the individual patient records in order to artificially elevate their performance. If they had done so, we would likely have seen larger differences between the arms.

It is interesting to compare the present study to other behavior change interventions to improve health care or health outcomes. Previous studies have shown that training alone has not generally been successful in changing malaria prescription practices even when coupled with the introduction of RDTs [32, 37, 38]. In contrast, innovative training methods can improve adherence to the results of malaria diagnostic testing, particularly for malaria-negative patients [32, 33]. This study provides evidence for an additional tool that can improve rational antimalarial use in the formal health sector. More broadly, our incentive strategy shows improvements similar in magnitude to other behavior change strategies such as opinion leaders (median absolute risk difference of 12 % for a broad range of health care outcomes [39] compared to a 9 % absolute risk difference in our study; Additional file 2) and audit and feedback (median absolute risk difference of 4 % [40]).

This is not a study of implementing P4P as a means of instigating sweeping reforms in the health system or health financing. We focused on a specific (although significant) problem and designed our incentives to impact that one area of concern. Our study only included government facilities, as these were the only facilities eligible for quarterly financing through the HSSF. In the relatively rural areas of our study, very few private health facilities exist, especially those with diagnostic capacity. Therefore, this criterion did not exclude many eligible facilities. However, we speculate that inclusion of private for-profit clinics in a facility-based incentive program might show even better results than in government facilities, since such facilities are profit-driven and may be highly motivated by the opportunity to earn additional resources that could be invested in their business. Although all of our study facilities had the capacity for microscopic diagnosis of malaria, implementation of RDT diagnosis could greatly expand the scope of facilities eligible to benefit from such a program. However, it has been observed that incentive programs are less successful when health care workers do not feel that they have the tools necessary to achieve their targets. If supplies of RDTs are erratic, this could seriously undermine the extrinsic motivation intended within an incentive scheme.

This work adds to the current literature in several important dimensions across multiple disciplines. First, it contributes new knowledge in the area of performance-based incentives, an area that has generated much interest but suffers from a lack of rigorous evidence in developing countries. It is the only study of which we are aware that has a truly experimental design for testing P4P incentives in sub-Saharan Africa. Our pay for performance program was also novel in two important respects. We focused on quality of care rather than volume of services and we used facility-directed rather than provider-directed incentives. Second, ours is a completely new approach to tackling the problem of overuse of antimalarials and improving malaria case management. Other studies have tested training interventions, introduction of new diagnostic tools and community education to improve adherence to the WHO policy of parasitological diagnosis before treatment, but this is the first report of using incentives to improve adherence to the policy. Finally, the results can inform quality improvement and behavior change interventions in health care delivery more broadly.

## Conclusions

This is the first experimental evidence of the impact of pay for performance incentives in sub-Saharan Africa. We demonstrate that facility-based incentives, rather than personal incentives paid to providers, were able to promote behavior change. This is particularly important in a resource-constrained system, since a facility-based approach allows funds to be re-invested in health infrastructure and enhances the sustainability and benefit to the patients. We observed that intervention facilities invested in infrastructure and significantly improved or expanded the services they were able to provide. In addition, the monetary value of the incentives in our program was designed to be offset by the money saved through reduced wastage of expensive ACTs.

Our study contributes to the small but growing body of evidence describing the usefulness and limitations of performance-based incentives in developing countries. It suggests that institutional incentives may provide extrinsic motivation for behavior change and enhance or sustain the effects of training, but this change may occur relatively slowly. A deeper understanding is required of the trade-offs between institutional incentives that may enhance health care infrastructure and service delivery versus individual incentives that may produce larger results in a shorter time period.

## Additional files

Additional file 1:
**Table of intracluster correlation coefficients (ICCs) for main study endpoints at month 12.** (PDF 27 kb) Additional file 2:
**A brief summary of estimating risk difference in our study for comparing to risk differences calculated for other behavior change interventions.** (DOCX 143 kb) Additional file 3:
**Full mixed-effects logistic regression results of AL use by malaria status over quarters and by age category.** (PDF 70 kb)

## Abbreviations

ACT: Artemisinin-based combination therapy
AL: Artemether-lumefantrine
AMPATH: Academic Model Providing Access to Healthcare
ANOVA: Analysis of variance
DHMT: District Health Management Team
HSSF: Health Sector Services Fund
ICC: Intracluster correlation coefficient
IRR: Incidence rate ratio
KSh: Kenya shilling
NIAID: National Institute of Allergy and Infectious Diseases
NIH: National Institutes of Health
OR: Odds ratio
P4P: Pay for performance
RDT: Rapid diagnostic test
USD: United States dollar
WHO: World Health Organization

## Footnotes

1 The facilities included in our study are located in what are now known as Busia County, Bungoma County, Uasin Gishu County, Elgeyo Marakwet County and Baringo County
