# Supplementary material for: Using pay for performance incentives (P4P) to improve management of suspected malaria fevers in rural Kenya: a cluster randomized controlled trial
Source: BMC Med. 2015 Oct 16;13:268. doi: 10.1186/s12916-015-0497-y (PMC4608124; doi:10.1186/s12916-015-0497-y)
Supplement: Additional file 1: — Table of intracluster correlation coefficients (ICCs) for main study endpoints at month 12. (PDF 27 kb) [file 12916_2015_497_MOESM1_ESM.pdf]

Table S2

Intraclass Correlation Coefficients

Population	Model	ICC	95% CI	
Malaria (-)	Null	0.416	0.218	0.644
Malaria (+)	Null	0.0802	0.00867	0.465
Malaria (-)	Unadjusted	0.413	0.217	0.642
Malaria (+)	Unadjusted	0.0724	0.00654	0.481
Malaria (-)	Adjusted	0.152	0.0498	0.380
Malaria (+)	Adjusted	0.000000314	0.000000000	1
