# Supplementary material for: Using pay for performance incentives (P4P) to improve management of suspected malaria fevers in rural Kenya: a cluster randomized controlled trial
Source: BMC Med. 2015 Oct 16;13:268. doi: 10.1186/s12916-015-0497-y (PMC4608124; doi:10.1186/s12916-015-0497-y)
Supplement: Additional file 2: — A brief summary of estimating risk difference in our study for comparing to risk differences calculated for other behavior change interventions. (DOCX 143 kb) [file 12916_2015_497_MOESM2_ESM.docx]

Menya Supplementary File 1:

We decided to estimate adjusted risk difference in our study as a means to make a crude comparison to other published works describing behavior change interventions targeted toward health worker behaviors.

Baseline-adjusted risk difference gives a more intuitive ‘difference in differences’ interpretation of the outcome than the ratio of odds ratios from the mixed effects model. However, it requires making assumptions about the levels of other covariates in the model when calculating risks and also does not allow for inclusion of facility random effects. Therefore it is an imperfect tool for this analysis, but does provide at least a point of comparison.

We report point estimates, delta-method standard errors, and 95% confidence intervals for calculations of differences in risk differences computed using Stata 13, xtlogit, margins, and nlcom commands drawing heavily from methodology of Norton et al [1]. Reported here are the baseline-adjusted risk differences which are the differences in the differences between mean predicted probabilities at baseline and quarter 4 for the intervention and control group. Predicted probabilities are computed for the unadjusted model using the (admittedly poor) assumption that the value for random effects are 0.

Table S4

| **Adjusted Risk Differences: Mixed effects logit (Unadjusted Model)** | | | | | | | | | | | |  | |  |
| --- | --- | --- | --- | --- | --- | --- | --- | --- | --- | --- | --- | --- | --- | --- |
|  | **Difference in ARDs** | | | | **RD Baseline** | | | | **RD Quarter 4** | | | | | |
| **AL given to malaria negative patients** | RD | Lower CI | Upper CI | P-value | RD - BL | Lower CI (1) | Upper CI (1) | P-value (1) | RD- Q4 | Lower CI | Upper CI | | P-value | |
| All ages | -0.090 | -0.217 | 0.038 | 0.168 | 0.068 | -0.108 | 0.244 | 0.448 | -0.022 | -0.081 | 0.038 | | 0.474 | |
| Ages 1-5 years | -0.078 | -0.229 | 0.074 | 0.315 | 0.061 | -0.145 | 0.266 | 0.614 | -0.017 | -0.083 | 0.049 | | 0.564 | |
| Ages 6+ years | -0.093 | -0.218 | 0.032 | 0.147 | 0.064 | -0.102 | 0.231 | 0.348 | -0.028 | -0.087 | 0.031 | | 0.447 | |
| High transmission | -0.111 | -0.256 | 0.035 | 0.136 | 0.029 | -0.214 | 0.272 | 0.211 | -0.082 | -0.210 | 0.046 | | 0.814 | |
| Low transmission | -0.056 | -0.122 | 0.010 | 0.097 | 0.046 | -0.026 | 0.118 | 0.182 | -0.010 | -0.025 | 0.005 | | 0.212 | |
| **AL given to malaria positive patients** |  |  |  |  |  |  |  |  |  |  |  | |  | |
| All ages | -0.024 | -0.150 | 0.103 | 0.712 | 0.009 | -0.120 | 0.138 | 0.890 | -0.015 | -0.112 | 0.083 | | 0.767 | |
| Ages 1-5 years | -0.013 | -0.188 | 0.161 | 0.880 | 0.020 | -0.145 | 0.186 | 0.920 | 0.007 | -0.131 | 0.145 | | 0.809 | |
| Ages 6+ years | -0.020 | -0.206 | 0.167 | 0.837 | 0.064 | -0.102 | 0.231 | 0.628 | -0.028 | -0.087 | 0.031 | | 0.938 | |
| High transmission | -0.104 | -0.248 | 0.041 | 0.160 | 0.076 | -0.059 | 0.210 | 0.459 | -0.028 | -0.102 | 0.046 | | 0.270 | |
| Low transmission | 0.016 | -0.262 | 0.295 | 0.908 | -0.108 | -0.316 | 0.100 | 0.483 | -0.091 | -0.347 | 0.164 | | 0.310 | |

[1] Norton, Edward C., Morgen M. Miller, and Lawrence C. Kleinman. "Computing adjusted risk ratios and risk differences in Stata." Stata Journal 13.3 (2013): 492-509.
