# Supplementary material for: Using pay for performance incentives (P4P) to improve management of suspected malaria fevers in rural Kenya: a cluster randomized controlled trial
Source: BMC Med. 2015 Oct 16;13:268. doi: 10.1186/s12916-015-0497-y (PMC4608124; doi:10.1186/s12916-015-0497-y)
Supplement: Additional file 3: — Full mixed-effects logistic regression results of AL use by malaria status over quarters and by age category. (PDF 70 kb) [file 12916_2015_497_MOESM3_ESM.pdf]

Table S1: Mixed Effects Logistic Regression of AL Use by Malaria Status by Quarters and by age category

MALARIA NEGATIVE

	Unadjusted OR			Adjusted OR		
Age	**0-5 years**	**6 + years**	**All ages**	**0-5 years**	**6 + years**	**All ages**
Quarter 1	**0.515**	**0.522**	**0.520**	**0.502**	**0.519**	**0.514**
	**(0.330 - 0.802)**	**(0.324 - 0.841)**	**(0.377 - 0.718)**	**(0.321 - 0.785)**	**(0.322 - 0.837)**	**(0.371 - 0.711)**
Quarter 2	**0.576**	**0.437**	**0.516**	**0.611**	**0.448**	**0.531**
	**(0.373 - 0.891)**	**(0.272 - 0.701)**	**(0.377 - 0.707)**	**(0.393 - 0.949)**	**(0.277 - 0.723)**	**(0.385 - 0.733)**
Quarter 3	**0.314**	**0.238**	**0.285**	**0.320**	**0.240**	**0.284**
	**(0.195 - 0.505)**	**(0.140 - 0.404)**	**(0.201 - 0.403)**	**(0.198 - 0.516)**	**(0.141 - 0.409)**	**(0.199 - 0.404)**
Quarter 4	**0.368**	**0.496**	**0.436**	**0.360**	**0.496**	**0.422**
	**(0.237 - 0.571)**	**(0.315 - 0.780)**	**(0.319 - 0.596)**	**(0.232 - 0.561)**	**(0.315 - 0.782)**	**(0.308 - 0.579)**
Intervention facility	1.558	1.735	1.719	1.272	1.504	1.441
	(0.348 - 6.969)	(0.425 - 7.085)	(0.431 - 6.858)	(0.514 - 3.148)	(0.581 - 3.894)	(0.628 - 3.305)
Quarter 1 x Intervention	0.854	0.870	0.860	0.865	0.863	0.859
	(0.474 - 1.541)	(0.470 - 1.609)	(0.564 - 1.311)	(0.479 - 1.564)	(0.466 - 1.597)	(0.561 - 1.314)
Quarter 2 x Intervention	0.617	0.930	0.739	0.597	0.902	0.721
	(0.345 - 1.104)	(0.507 - 1.709)	(0.488 - 1.119)	(0.333 - 1.070)	(0.490 - 1.661)	(0.474 - 1.097)
Quarter 3 x Intervention	0.854	1.226	0.974	0.960	1.241	1.060
	(0.461 - 1.583)	(0.634 - 2.371)	(0.625 - 1.517)	(0.514 - 1.791)	(0.639 - 2.408)	(0.675 - 1.664)
Quarter 4 x Intervention	**0.433**	**0.275**	**0.341**	**0.468**	**0.275**	**0.364**
	**(0.236 - 0.793)**	**(0.146 - 0.519)**	**(0.221 - 0.526)**	**(0.254 - 0.861)**	**(0.145 - 0.520)**	**(0.235 - 0.565)**
RDT used				**0.660**	0.913	**0.773**
				**(0.466 - 0.935)**	(0.639 - 1.304)	**(0.602 - 0.992)**
Age <6						**1.263**
						**(1.116 - 1.430)**
Female				1.059	0.859	0.965
				(0.896 - 1.251)	(0.711 - 1.037)	(0.852 - 1.093)
High Transmission Area				**11.33**	**9.450**	**10.36**
				**(5.210 - 24.62)**	**(4.215 - 21.18)**	**(4.962 - 21.64)**
Mean monthly slides read (2012)				1.000	1.000	1.000
				(0.998 - 1.001)	(0.998 - 1.002)	(0.998 - 1.002)
Constant	**0.156**	**0.118**	**0.132**	**0.0552**	**0.0452**	**0.0415**
	**(0.0521 - 0.467)**	**(0.0419 - 0.333)**	**(0.0478 - 0.362)**	**(0.0178 - 0.171)**	**(0.0149 - 0.137)**	**(0.0155 - 0.111)**
Standard deviation: facility random effect	1.461	1.378	1.401	0.715	0.789	0.733
	(0.989 - 2.159)	(0.959 - 1.980)	(0.988 - 1.989)	(0.473 - 1.081)	(0.539 - 1.155)	(0.511 - 1.052)
ICC	0.394	0.366	0.374	0.135	0.159	0.140
	(0.229 - 0.586)	(0.219 - 0.544)	(0.229 - 0.546)	(0.0637 - 0.262)	(0.0810 - 0.288)	(0.0734 - 0.252)
Observations (n)	5,414	6,328	11,927	5,412	6,328	11,740
Number of Facilities	17	17	17	17	17	17

MALARIA POSITIVE

Unadjusted OR			Adjusted OR		
**0-5 years**	**6 + years**	**All ages**	**0-5 years**	**6 + years**	**All ages**
0.730	1.218	0.925	0.730	1.260	0.941
(0.390 - 1.364)	(0.636 - 2.335)	(0.593 - 1.441)	(0.391 - 1.364)	(0.656 - 2.417)	(0.602 - 1.472)
1.299	1.802	1.522	1.310	1.887	1.544
(0.677 - 2.491)	(0.929 - 3.495)	(0.963 - 2.405)	(0.682 - 2.515)	(0.969 - 3.673)	(0.973 - 2.450)
1.232	2.091	1.466	1.241	2.067	**1.591**
(0.654 - 2.321)	(1.081 - 4.044)	(0.936 - 2.295)	(0.656 - 2.348)	(1.065 - 4.013)	**(1.007 - 2.515)**
1.163	1.908	1.468	1.122	1.834	1.452
(0.606 - 2.230)	(0.987 - 3.688)	(0.929 - 2.317)	(0.584 - 2.155)	(0.949 - 3.544)	(0.915 - 2.305)
1.119	0.970	1.046	1.246	0.938	1.067
(0.451 - 2.772)	(0.452 - 2.082)	(0.555 - 1.970)	(0.533 - 2.913)	(0.446 - 1.975)	(0.590 - 1.928)
1.343	1.679	1.467	1.323	1.597	1.480
(0.535 - 3.370)	(0.690 - 4.086)	(0.783 - 2.747)	(0.527 - 3.323)	(0.654 - 3.903)	(0.786 - 2.786)
0.876	0.619	0.692	0.889	0.567	0.691
(0.343 - 2.236)	(0.259 - 1.478)	(0.370 - 1.293)	(0.348 - 2.270)	(0.237 - 1.358)	(0.368 - 1.297)
0.679	0.821	0.811	0.798	0.873	0.849
(0.271 - 1.704)	(0.344 - 1.957)	(0.437 - 1.505)	(0.313 - 2.034)	(0.364 - 2.090)	(0.452 - 1.595)
0.931	0.885	0.879	0.978	0.873	0.914
(0.349 - 2.486)	(0.359 - 2.181)	(0.457 - 1.690)	(0.365 - 2.618)	(0.354 - 2.155)	(0.473 - 1.768)
			0.724	0.775	0.764
			(0.461 - 1.137)	(0.520 - 1.156)	(0.565 - 1.033)
					1.028
					(0.858 - 1.231)
			1.226	**0.764**	0.970
			(0.952 - 1.579)	**(0.596 - 0.980)**	(0.814 - 1.156)
			**2.045**	**1.708**	**1.737**
			**(1.253 - 3.336)**	**(1.273 - 2.292)**	**(1.238 - 2.437)**
			**1.001**	1.000	1.000
			**(1.000 - 1.002)**	(0.999 - 1.001)	(0.999 - 1.001)
**2.982**	**1.973**	**2.464**	1.136	1.801	1.578
**(1.587 - 5.602)**	**(1.112 - 3.500)**	**(1.559 - 3.894)**	(0.461 - 2.802)	(0.896 - 3.620)	(0.851 - 2.927)
0.478	0.270	0.372	0.313	0.129	0.254
(0.295 - 0.774)	(0.146 - 0.501)	(0.240 - 0.577)	(0.162 - 0.607)	(0.0332 - 0.498)	(0.149 - 0.435)
0.0649	0.0217	0.0404	0.0289	0.00500	0.0193
(0.0258 - 0.154)	(0.00641 - 0.071)	(0.0172 - 0.092)	(0.00787 - 0.101)	(0.000335 - 0.0700)	(0.00667 - 0.054)
1,458	1,475	2,972	1,458	1,474	2,932
16	17	17	16	17	17
